# Cough-Inducing Method Using a Tartaric Acid Nebulizer for Patients with Silent Aspiration

**DOI:** 10.1007/s00455-021-10313-4

**Published:** 2021-05-11

**Authors:** Tomohisa Ohno, Naomi Tanaka, Mariko Fujimori, Keishi Okamoto, Satoe Hagiwara, Kyoko Hojo, Takashi Shigematsu, Takafumi Sugi, Hideaki Kanazawa, Kenjiro Kunieda, Ichiro Fujishima

**Affiliations:** 1 Department of Dentistry, Hamamatsu City Rehabilitation Hospital, Hamamatsu, Japan; 2 Department of Nursing, Hamamatsu City Rehabilitation Hospital, Hamamatsu, Japan; 3 Department of Nursing, Seirei Mikatahara General Hospital, Hamamatsu, Japan; 4 Department of Rehabilitation, Hamamatsu City Rehabilitation Hospital, Hamamatsu, Japan; 5 Department of Rehabilitation Medicine, Hamamatsu City Rehabilitation Hospital, 1-6-1 Wagokita, Naka-ku, Hamamatsu, Shizuoka 433-8511 Japan; 6 Department of Neurology, Gifu University Graduate School of Medicine, Gifu, Japan

**Keywords:** Aspiration, Cough, Deglutition disorders, Tartaric acid

## Abstract

The tartaric acid nebulizer is a well-known cough test to evaluate cough function. This study aimed to evaluate the effectiveness of a cough-inducing method using tartaric acid (CiTA). Patients with dysphagia examined by videofluoroscopic examination of swallowing (VF) at a single institution from May 2017 to August 2017 were included in this retrospective observational study. Although undergoing VF, patients who had aspirated without reflexively coughing or who had coughed insufficiently, were instructed to cough voluntarily. Patients who could not cough voluntarily or had expectorated insufficiently underwent the CiTA method. The rate of cough induction and the effectiveness of expectoration using the CiTA method were evaluated. One hundred fifty-four patients (mean age 69.2 ± 16.8 years) were evaluated. Eighty-seven patients aspirated during VF. Of those patients, 15 were able to expectorate via the cough reflex, 18 were able to expectorate with a voluntary cough, and 12 required suctioning for removal of aspirated material. The remaining 42 patients underwent the CiTA method. Thirty-eight patients (90.4%) could reflexively cough, and 30 (71.4%) could expectorate the aspirated material. This novel method, CiTA, was effective for cough induction in patients with dysphagia, especially for those with silent aspiration.

## Introduction

Silent aspiration in patients with dysphagia could lead to aspiration pneumonia. Management of silent aspiration is important for oral intake and life prognosis [1]. Silent aspiration includes two aspects, one is an aspiration of secretion, such as saliva or gastric contents by regurgitation, especially during sleep, and the other is an aspiration of food related to a meal [1, 2]. Because silent aspiration is the aspiration of material without a reflexive cough response, it is difficult to determine whether silent aspiration has occurred based on clinical observation alone. In dysphagia patients, silent aspiration related to meals is a serious issue. Silent aspiration is caused by decreased sensation of the airways, especially the laryngeal mucosa that is innervated by the superior laryngeal nerve [1]. A decreased cough reflex has been reported to be caused by aging [3] and cerebrovascular disease [4], especially cerebrovascular disease in the acute stage [5]. A decreased cough reflex could lead to aspiration pneumonia [6, 7].

To decrease the risk of aspiration pneumonia, it is necessary to establish a method for detecting the risk of silent aspiration and a method for treating silent aspiration in patients with dysphagia. Videofluoroscopic examination of swallowing (VF) can detect silent aspiration. Garon et al. reported that about half of the patients with dysphagia aspirated during VF, and about half of them aspirated silently [8, 9]. However, there are limitations regarding where and when VF can be performed because this examination exposes the patient and the surroundings to radiation and requires specific preparations regarding time and place. The cough test using citric acid or tartaric acid is used as an evaluation method for cough function. The test is less commonly used as a screening test for silent aspiration in patients with dysphagia in actual clinical situations [10–12]. For the cough test, a solution of citric acid or tartaric acid is placed in a nebulizer and the patient inhales the solution as a micro aerosol. Although this test has been used as an evaluation of cough function and as a screening test for silent aspiration, to our knowledge, no studies have evaluated the use of this method for inducing cough.

The widely practiced compensatory strategies for patients with dysphagia related to meals are positioning strategies and modified diet; however, these are not directly effective strategies for a patient who has silent aspiration. There are some treatment methods for aspiration or suspected silent aspiration. The first is voluntary cough. During VF, if a reflexive cough is not induced when the patient with dysphagia aspirates a barium-contained modified diet (i.e., silent aspiration), the examiner instructs the patient to voluntarily cough to expectorate the aspirated food. The second is pulmonary rehabilitation, such as support for expectoration of secretions. The third is intratracheal suction. These are common methods for treating aspirations. However, it may be difficult for patients with dysphagia to cough voluntarily, especially those who have cerebrovascular disease [10]. Pulmonary rehabilitation includes respiratory muscle training and breathing exercises [13] to enhance swallowing-respiration coordination, assist cough ability, and aid in airway clearance. It is clinically effective, but may be difficult to implement immediately. Furthermore, pulmonary rehabilitation is conducted by professionals, such as physical therapists, and requires additional time. Intratracheal suction is quite useful but invasive, placing a heavy burden on patients. Therefore, it is necessary to develop a simple and less-invasive cough reflex induction method that can be performed rapidly when voluntary coughing is not possible. In addition, a less-invasive cough reflex induction method may be useful not only during VF, but also during usual meals. Although the method involves a disruption of the natural mealtime environment, it may lead to decreased risk of aspiration pneumonia.

Previously, tartaric acid nebulizer was used primarily as a cough test to evaluate the cough function by inducing reflexive cough. In our hospital, we have begun to use tartaric acid nebulizer as a cough-inducing method for patients with silent aspiration with dysphagia. This study presents a novel method using a tartaric acid nebulizer to induce cough that is simple and less-invasive for silent aspiration during VF, and also for patients with suspected silent aspiration. This may lead to the prevention of aspiration pneumonia. To our knowledge, there are no reports describing this method and its effectiveness.

In this study, the effectiveness of the cough-inducing method with a tartaric acid nebulizer (CiTA) during VF was evaluated based on the hypothesis that the CiTA method could induce reflexive cough and increase the effectiveness of expectoration. In addition, the frequency of silent aspiration during VF and related factors was investigated.

## Methods

This single-center, retrospective observational study was conducted at the Hamamatsu City Rehabilitation Hospital from May 2017 to August 2017. The consents were obtained by the opt-out method. The study was conducted in accordance with the standards of the Declaration of Helsinki. This study was approved by the Ethics Committee of the Hamamatsu City Rehabilitation Hospital (protocol approval no. 17-04, approval date February 9, 2017).

### Participants

The participants were inpatients with dysphagia. Sequential patients who were examined by VF during the study period were evaluated. The necessity of VF was decided by the attending physician. The exclusion criteria were those who could not be evaluated at the pharyngeal stage using VF because the bolus could not transit the oral cavity.

### Baseline Characteristics

The patients’ medical records were evaluated retrospectively. The baseline characteristics of the patients included in the analysis were age, sex, main disease for admission, history of aspiration pneumonia, past recurrence of cerebrovascular disease, history of respiratory diseases except aspiration pneumonia, and the Food Intake LEVEL scale (FILS) [14]. The FILS is used to assess the severity of dysphagia as follows: a FILS score of 1–3 indicates no oral intake, a FILS score of 4–6 indicates oral intake and alternative nutrition, and an FILS score of 7–10 indicates oral intake alone.

### Evaluation During VF

During VF, barium gelatin jelly and thickened barium water were used as test foods. The amount of test food was approximately 3–5 g and was not clearly defined. The collected VF data included the presence of aspiration, the Penetration–Aspiration (PA) scale [15], and expectoration by cough in patients with aspiration. The 8-point PA scale measures the severity of airway invasion during swallowing. A score of 1 indicates no risk of aspiration, scores of 2–5 indicate moderate risk of aspiration, and scores of 6–8 indicate severe risk of aspiration. First, patients who were able to produce expectorate with a reflexive cough were counted. If a patient was observed to have silent aspiration on VF or had reflexively coughed insufficiently, we instructed the patient to cough hard voluntarily.

If the patient was unable to cough voluntarily or had expectorated insufficiently, the CiTA method was applied. A nebulizer that included a 10% solution of *l*-tartaric acid was used to induce the cough reflex. The cough induction rate and the rate of the effectiveness of expectoration using the CiTA method were evaluated. If a large amount of barium containing food remained in the trachea and the patient was unable to expectorate, intratracheal suction was performed.

### Statistical Analysis

Statistical analyses were performed to determine whether patients could expectorate based on the CiTA method. The values were compared using the Mann–Whitney U test and Fisher’s exact test. The threshold value for rejecting the null hypothesis was P < 0.05. All statistical analyses were performed using IBM SPSS statistics version.26 software (IBM Japan Corp., Tokyo, Japan).

## Results

### Baseline Characteristics of the Patients

One hundred and fifty-four patients were included. No patients were excluded. Table 1 shows the characteristics of the patients. The mean age of the patients was over 65 years. More than half of the patients had cerebrovascular disease, and 49 (31.8%) had a history of aspiration pneumonia. The median FILS was 6; therefore, many patients with moderate dysphagia were included in this study.

**Table.1:** Baseline characteristics of the patients

Variables	All (*n* = 154)	
Age, mean years ± SD	69.2 ± 16.8	
Male, *n* (%)	97	(63.0%)
Main disease, *n* (%)		
Cerebrovascular disease	91	(59.0%)
Neuromuscular disease	14	(9.1%)
Disuse syndrome	13	(8.4%)
Brain injury	12	(7.9%)
Aspiration pneumonia	6	(4.0%)
Brain tumor	6	(4.0%)
Hip fracture	4	(2.7%)
Others	10	(6.6%)
History of aspiration pneumonia, *n* (%)	49	(31.9%)
Past recurrence of cerebrovascular disease, *n* (%)	36	(23.4%)
History of respiratory disease except aspiration pneumonia, *n* (%)	14	(9.1%)
FILS, score (IQR)	6	(3–8)

*FILS* food intake LEVEL Scale, *IQR* interquartile range

### Evaluation During VF Regarding Aspiration and the Penetration–Aspiration scale

**Fig. 1:**
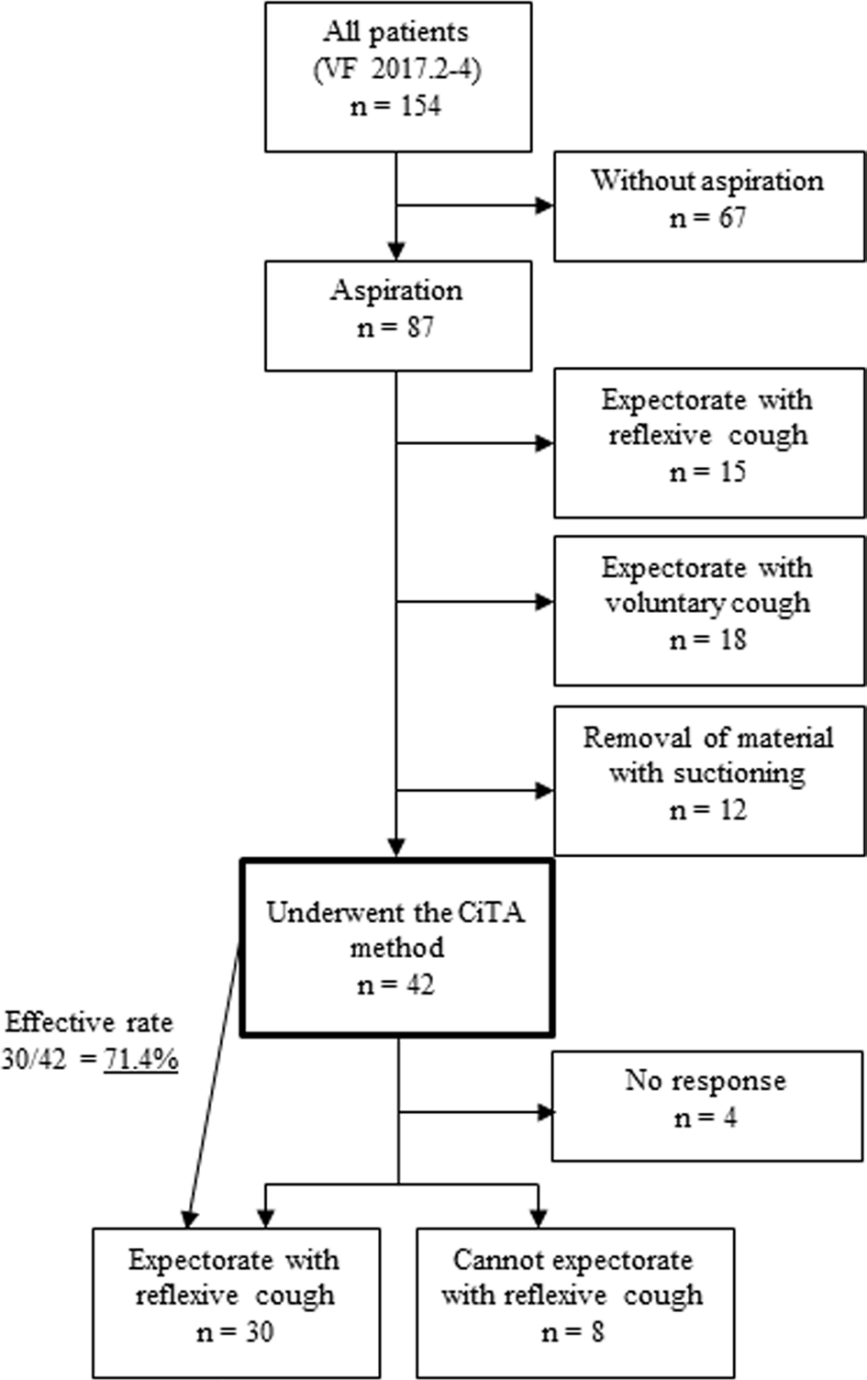
Results and flow diagram of this study. *VF* videofluoroscopic examination of swallowing, *CiTA* cough-inducing method using a tartaric acid nebulizer

Figure 1 and Table 2 present the main results of this study. Eighty-seven patients (56.5%) exhibited aspiration during VF. Approximately half of the patients (46.8%) were at point 8 on the PA scale, which indicates silent aspiration. Of the 87 patients, 15 were able to expectorate by reflexive cough, 18 were able to expectorate by voluntary cough, and 12 required removal of aspirated food by suctioning. The remaining 42 patients underwent the CiTA method.

**Table 2:** Findings during videofluoroscopic examination

	*n* (%)	
All patients	154	
Without aspiration	67	(43.5%)
With aspiration	87	(56.5%)
PA scale		
Score 1	59	(38.3%)
Score 2	4	(2.6%)
Score 3	1	(0.6%)
Score 4	3	(1.9%)
Score 5	0	(0%)
Score 6	1	(0.6%)
Score 7	14	(9.1%)
Score 8	72	(46.8%)
Patients with aspiration	87	
Expectorate by reflexive cough	15	(17.2%)
Silent aspiration	72	(82.8%)
Expectorate by voluntary cough	18	(20.7%)
Removed by suctioning	12	(16.7%)
Patients who underwent the CiTA method	42	
No response	4	(9.5%)
With cough reflex	38	(90.5%)
Patients with cough reflex using tartaric acid	38	
Expectorate by reflexive cough	30	(78.9%)
Cannot expectorate by reflexive cough	8	(21.1%)

*PA scale* Penetration–Aspiration scale, *CiTA* cough-inducing method using a tartaric acid nebulizer

### Effectiveness of the CiTA method

Of the 42 patients, 38 (90.4%) had a cough reflex that was induced by the CiTA method. Thirty patients (71.4%) were able to expectorate the aspirated food. Baseline characteristics of the patients were compared statistically between those who could expectorate (30 patients) and those who could not expectorate (12 patients). As a result, there was a significant difference in the history of aspiration pneumonia (Table 3). In addition, no patients experienced any adverse event, such as laryngospasm by undergoing the CiTA method.

**Table 3:** Results and characteristics of patients using the CiTA method (*n* = 42)

Variables	Can expectorate (*n* = 30)	Cannot expectorate (*n* = 12)	*p* value
Age, years	74.2 ± 11.5	77.7 ± 10.1	0.369
Male, *n* (%)	20 (66.7%)	9 (75.0%)	0.722
Main disease, *n* (%)			0.207
Cerebrovascular disease	18 (60.0%)	6 (50.0%)	
Neuromuscular disease	3 (10.0%)	0	
Disuse syndrome	3 (10.0%)	0	
Brain injury	3 (10.0%)	3 (25.0%)	
Aspiration pneumonia	0	3 (25.0%)	
Brain tumor	2 (6.7%)	0	
Hip fracture	0	0	
Others	1 (3.3%)	0	
History of aspiration pneumonia, *n* (%)	8 (26.7%)	8 (66.7%)	0.032*
Past recurrence of cerebrovascular disease, *n* (%)	8 (26.7%)	6 (50.0%)	0.169
History of respiratory disease without aspiration pneumonia, *n* (%)	3 (10.0%)	2 (16.7%)	0.613
FILS, score	7 (4–8)	5 (3–7)	0.198
PA scale, *n* (%)			1.000
Score 1–7	0	0	
Score 8	30 (100%)	12 (100%)	

*FILS* Food Intake LEVEL Scale, *PA scale* Penetration–Aspiration scale

*p < 0.05

## Discussion

In this study, the tartaric acid nebulizer was used not as a cough test, but as a novel, less-invasive cough reflex-inducing method (CiTA) to expectorate aspirated food. The hypothesis was that CiTA could induce reflexive cough and increase the effectiveness of expectoration. The data suggest that CiTA was able to induce cough in many patients with silent aspiration, although the effectiveness rate of expectoration was significantly low in patients with recurrent pneumonia. The results of this study suggest that CiTA is a useful method for patients with silent aspiration.

In this study, a 10% solution of *l*-tartaric acid was used as the stimulant to produce the cough. Most cough tests have used a 20% tartaric acid solution [10, 16–19], which may be appropriate for single use as a screening test. However, this concentration may be too strong for use as a repetitive cough-inducing method during daily meals. A cough attack and laryngeal spasm may occur, which may interfere with swallowing training. Therefore, this study used half the concentration of tartaric acid used in the cough test. Because the inducing rate and the effectiveness rate were high, the concentration of tartaric acid was thought to be appropriate.

The most important result was that the CiTA was able to induce a sufficient cough reflex to expectorate aspirated food in patients who could not cough voluntarily. In this study, the expectoration rate was over 70% in patients with dysphagia who had difficulty coughing voluntarily or in those with insufficient cough. This study included patients with dysphagia who had aspirated silently during VF. There are also patients with dysphagia who have silent aspiration of food during their daily meals. To decrease the risk of aspiration, voluntary cough has been used clinically in Japan for patients with dysphagia who are suspected of silent aspiration. In addition, the supraglottic swallow method requires a voluntary cough or exhalation in the last procedure to decrease the risk of aspiration [20]. However, voluntary cough may not be possible if cognitive function is impaired. In addition, dysphagia may be caused by various diseases [21], including diseases that reduce cognitive function, such as cerebrovascular diseases and dementia [22]. The CiTA method is simple, and may be less dependent on the cognitive function impaired by dementia, aphasia, and other communication disorders. Therefore, CiTA may be effective in patients with cognitive impairment. Further study on the effectiveness of CiTA in patients with dementia is necessary.

The second important result in this study is the high cough induction rate using the CiTA method. The reflexive cough induction rate was over 90% in patients with dysphagia who had difficulty with voluntary coughing or insufficient cough. Because the risk of aspiration is high in patients with dysphagia, various methods, such as regulation of posture, modification of the diet, “think swallow” (swallow with sufficient awareness of swallowing) [23], and supraglottic swallow are applied to decrease the risk of aspiration [20]. Unfortunately, the risk of aspiration cannot be reduced to zero with these methods. Therefore, it is also necessary to ensure that the patient has expectorated, and that the material has been removed, even if they have aspirated. This may reduce the risk of aspiration pneumonia, atelectasis, and choking. There have been few treatment methods for those who have aspirated. The most common method, intratracheal suction, is irritating and places a heavy burden on the patient. The cough reflex is caused by stimulation of nerve receptors in the vagal terminal branches located in the larynx and bronchial bifurcation. Stimuli include airway inflammation and mechanical and chemical stimuli [24]. The main purpose of intratracheal suction is not to cause the cough reflex but to remove aspirated material. Intratracheal suction provides mechanical stimulus to the nerve receptors, strongly stimulates the cough reflex, and is invasive. Furthermore, suction pressure that is too high and continuous suction both increase the risk of mucosal injury [25]. Other methods used to treat aspiration are pulmonary rehabilitation techniques for expectorate aspiration, which include huffing [26] and the active cycle breathing technique [27]. However, it may be difficult to apply these techniques immediately because these methods require professional skills, and patients must be able to follow instructions. The CiTA method is not mechanical; rather, it is a chemical stimulation that places a relatively small burden on the patient and the care providers. Minimal training is required, and CiTA can be immediately performed as needed. Anyone can use CiTA, including medical professionals, such as nurses, doctors, dentists, and care givers, as well as the patient, and there is no limitation regarding specialty care. Notably, CiTA can be used anywhere, such as in hospital wards, at home, or in a nursing home. On the other hand, the CiTA induces a reflexive cough. Therefore, diseases and symptoms that may be exacerbated by cough, such as heart failure, pulmonary edema, asthma, and pneumothorax, may be considered contraindications.

The reason for the low effectiveness rate of the CiTA method in patients with a history of aspiration pneumonia, especially recurrent cases, is that the threshold for inducing the cough reflex may have increased. Niimi et al. reported that patients with recurrent pneumonia have a high threshold for the cough reflex [28]. On the other hand, in our study, there were eight patients who benefited from the CiTA method even with their history of pneumonia. Considering this finding, the CiTA method may also be useful for patients with a history of pneumonia. This method is slightly less effective for patients with a history of aspiration pneumonia.

This study has several limitations. First, because this study was conducted at one institution, it is unknown whether the same result will be obtained at other institutions. Wakasugi et al. have reported a sensitivity of 0.86 for a cough test using a citric acid nebulizer in patients with silent aspiration [11]. Addington et al. have reported that the tartaric acid nebulizer cough test could induce a reflexive cough in 360 of 400 patients (90%) [10]. This study revealed a 90.4% rate of reflexive cough induction using the CiTA method. Because the rate of cough reflex induction at other institutions is similar to the result of this study, the generalization bias may not be a concern. Second, this study did not examine whether citric acid [3–5, 11, 29] and capsaicin [30, 31], which are used as stimulants for other cough tests, could be used as a cough-inducing method. Further research is also needed regarding the timing and frequency of application, safety, familiarity, and activation effects of long-term use of the CiTA method.

## Conclusion

The CiTA is a novel method for inducing reflexive cough. This method was used as a reflexive cough-inducing method, not as a cough test or screening test. We used CiTA during VF for dysphagia patients with silent aspiration and difficulty with voluntary cough. The CiTA was able to induce reflexive cough in 90.4% of the patients, and 71.4% of the patients could expectorate the aspirated food. The CiTA was effective for cough induction in patients with dysphagia, especially for those with silent aspiration. This method may be useful for cough induction in patients with silent aspiration.
